# Differential Abilities of Mammalian Cathelicidins to Inhibit Bacterial Biofilm Formation and Promote Multifaceted Immune Functions of Neutrophils

**DOI:** 10.3390/ijms21051871

**Published:** 2020-03-09

**Authors:** Fang Xie, Yanan Zan, Xinyuan Zhang, Huihui Zhang, Mingjie Jin, Wanjiang Zhang, Yueling Zhang, Siguo Liu

**Affiliations:** 1 State Key Laboratory of Veterinary Biotechnology, Harbin Veterinary Research Institute, Chinese Academy of Agricultural Sciences, Harbin 150069, China; xiefang@caas.cn (F.X.); zanyananhsy@163.com (Y.Z.); zxycinya@126.com (X.Z.); zxh075420@163.com (H.Z.); czandjmj@163.com (M.J.); zhangwanjiang@caas.cn (W.Z.); zhangyueling@caas.cn (Y.Z.); 2 College of Veterinary Medicine, Inner Mongolia Agricultural University, Huhehaote 010000, China; 3 College of Veterinary Medicine, Northeast Agricultural University, Harbin 150069, China

**Keywords:** antimicrobial peptide, cathelicidins, bactericidal activity, antibiofilm, immunomodulation, neutrophils, chemotaxis, extracellular traps, *Pseudomonas aeruginosa*

## Abstract

Mammalian cathelicidins act as the potent microbicidal molecules for controlling bacterial infection, and are considered promising alternatives to traditional antibiotics. Their ability to modulate host immune responses, as well as their bactericidal activities, is essential for therapeutic interventions. In this study, we compared the bactericidal activities, antibiofilm activities and immune-modulatory properties of cathelicidins BMAP-27, BMAP-34, mCRAMP, and LL-37, and evaluated the therapeutic efficacy of the combination of BMAP-27 and LL-37 using a mouse pulmonary infection model. Our results showed that all of the four cathelicidins effectively killed bacteria via rapid induction of membrane permeabilization, and BMAP-27 exhibited the most excellent bactericidal activity against diverse bacterial pathogens. BMAP-27, mCRAMP, and LL-37 effectively inhibited biofilm formation, while BMAP-34, mCRAMP and LL-37 exerted immunomodulatory functions with varying degrees of efficacy by stimulating the chemotaxis of neutrophils, inducing the production of reactive oxygen species, and facilitating the formation of neutrophil extracellular traps. Of note, the combination of BMAP-27 and LL-37 effectively enhanced the clearance of *Pseudomonas aeruginosa* and reduced the organ injury *in vivo*. Together, these findings highlight that identifying the appropriate synergistic combination of mammalian cathelicidins with different beneficial properties may be an effective strategy against bacterial infection.

## 1. Introduction

Overuse of antibiotics in humans and animals contributes to worldwide spread of multidrug-resistant (MDR) strains, such as so-called ESKAPE (*Enterococcus faecium*, *Staphylococcus aureus*, *Klebsiella pneumoniae*, *Acinetobacter baumannii*, *Pseudomonas aeruginosa*, and *Enterobacter species*) pathogens [1]. The ability of biofilm formation makes these pathogens even more difficult to treat [2], highlighting the need for new therapeutic strategies to tackle the current grim constitution.

Mammalian cathelicidins constitute an important family of antimicrobial peptides (AMPs) and are essential defense molecules against microbial infections [3]. They are synthesized as inactive precursors, including a highly conserved N-terminal region known as the cathelin domain, and a C-terminal domain with a varied sequence and length [4]. The precursors are temporally stored within the specific granules of neutrophils. Upon activation, the C-terminal region known as the mature peptide with biological activity is cleaved from the cathelin domain by proteinases or elastases [5,6]. Among the mammalian cathelicidins, human cathelicidin LL-37 not only has the bactericidal activity but also modulates the immune responses [7,8]. Its murine ortholog, mCRAMP, is also an important member of this family, as evidenced by *in vivo* studies documenting that *Cramp*-deficient mice are more susceptible to bacterial infection than wild-type mice [9]. In addition, although the immune-modulatory properties remain not well understood, bovine cathelicidin peptides BMAP-27 and BMAP-34 have been shown to efficiently inhibit the growth of pathogenic bacteria [10]. Mammalian cathelicidins are highlighted as promising alternatives to traditional antibiotics for the treatment of bacterial infection [3].

*In vivo* functional analysis of AMPs in *Drosophila* has found that they could act synergistically, thus jointly contributing to defense against bacterial infection [11]. This example of additive cooperation of AMPs provides valuable information for considering future therapeutic strategies regarding the combination of AMPs. Notably, the therapeutic efficacy of AMPs was evaluated in the clinical context not only for their direct antibacterial activity, but also the indirect immunomodulatory activity to promote the clearance of bacterial infection [3,12]. The aims of this study were to compare the antibacterial and antibiofilm activities of the four cathelicidins BMAP-27, BMAP-34, mCRAMP, and LL-37, to determine their immunomodulatory activity on neutrophils, and to evaluate the therapeutic efficacy of the combination of BMAP-27 and LL-37 against *P. aeruginosa* infection. Overall, this study demonstrates that the combination of the outstanding bactericidal and antibiofilm property of BMAP-27 with the immune promotion effect of LL-37 is a promising therapeutic strategy to enhance the clearance of *P. aeruginosa* infection.

## 2. Results

### 2.1. BMAP-27, BMAP-34, mCRAMP, and LL-37 Exhibit Differential Bactericidal Activities

**Figure 1:**
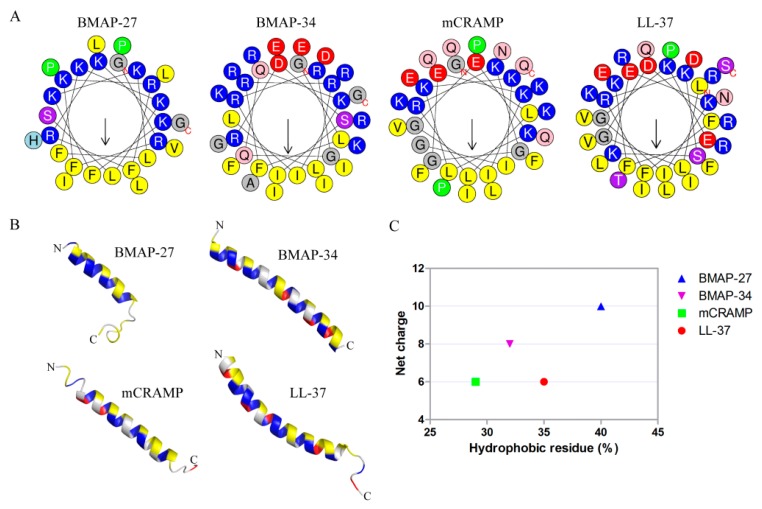
Structures and characteristics of mammalian cathelicidins. (**A**) Helical wheel depiction of mammalian cathelicidins. (**B**) Structures of mammalian cathelicidins. The 3D structures of BMAP-27 (PDB: 2KET) and LL-37 (PDB: 2K6O) were obtained from the Protein Data Bank. The 3D structures of BMAP-34 and mCRAMP were predicted using I-TASSER. (**C**) Values for net charge and percentage for hydrophobic amino acids of mammalian cathelicidins as obtained from the APD3 database.

The secondary structure of an antimicrobial peptide is greatly responsible for its bactericidal ability [13]. BMAP-27, BMAP-34, mCRAMP, and LL-37 all showed an α-helical conformation, as indicated by the helical wheel analysis (Figure 1A), as well as three-dimensional (3D) structural models (Figure 1B). BMAP-27 adopts the typical amphipathic α-helical structure, with hydrophobic and positively charged residues distributed on each side, while those of BMAP-34, mCRAMP, and LL-37 are more dispersed (Figure 1A,B). Additionally, the positive net charge and hydrophobicity of BMAP-27 are higher than those of other cathelicidins, while mCRAMP has less positive net charge and lower hydrophobicity (Figure 1C).

**Figure 2:**
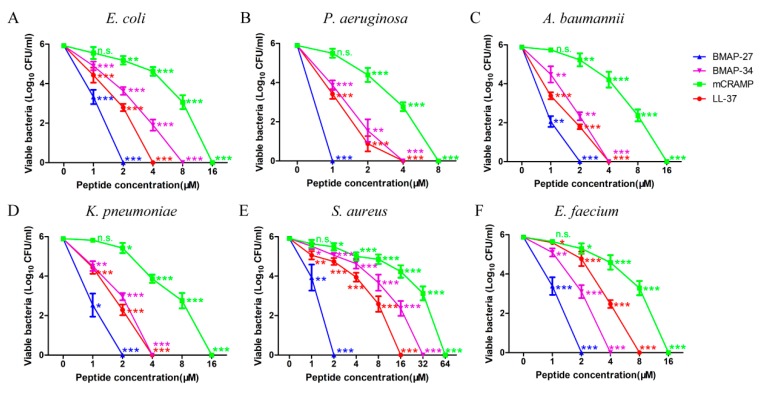
Mammalian cathelicidins effectively kill both Gram-negative and Gram-positive bacteria. Bactericidal activities of BMAP-27, BMAP-34, mCRAMP, and LL-37 on (**A**) *E. coli* ATCC 25922, (**B**) *P. aeruginosa* ATCC 27853, (**C**) *A. baumannii* ATCC 19606, (**D**) *K. pneumoniae* ATCC 700603, (**E**) *S. aureus* ATCC 29213, and (**F**) *E. faecium* ATCC 700221. Each strain was incubated with different peptides at varying concentrations for 2 h, and the number of bacterial colonies was calculated. Values represent the mean ± SD from three independent assays, * *p* < 0.05, ** *p* < 0.01, *** *p* < 0.001, n.s. = not significant.

The bactericidal activities of BMAP-27, BMAP-34, LL-37, and mCRAMP against Gram-negative and Gram-positive bacteria were subsequently measured. As shown in Figure 2, all of the four cathelicidins cause a concentration-dependent decline in the number of surviving bacteria following incubation in the presence of each peptide. The results showed that all of the four cathelicidins exerted efficient and broad-spectrum activities against Gram-negative and Gram-positive bacteria (Figure 2). Among them, BMAP-27 was the most effective, with minimal bactericidal concentrations (MBCs) between 1 and 2 μM for both Gram-negative bacteria and Gram-positive bacteria. The bactericidal activities of LL-37 and BMAP-34 were lower than those of BMAP-27, with MBCs from 4 to 8 μM for Gram-negative bacteria and 4 to 32 μM for Gram-positive bacteria. mCRAMP had reduced bactericidal activities compared with the other three peptides, with MBCs from 8 to 16 μM for Gram-negative bacteria and from 16 to 64 μM for Gram-positive bacteria.

### 2.2. BMAP-27, BMAP-34, mCRAMP, and LL-37 Kill Bacteria via Rapid Induction of Membrane Permeabilization

**Figure 3:**
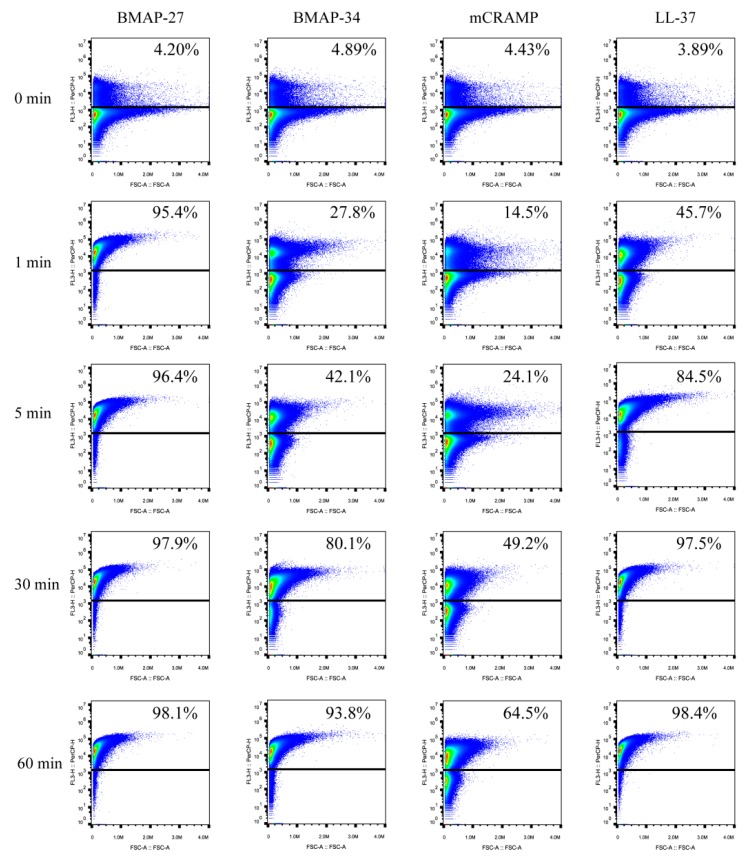
Mammalian cathelicidins rapidly penetrate the bacterial cell membrane. The membrane permeability of *E. coli* treated by 4 μM of BMAP-27, BMAP-34, mCRAMP, and LL-37 as measured by an increase in fluorescence intensity of PI using a flow cytometry assay. A time-course membrane permeability was observed within 1 h.

To explore the bactericidal rates of the four cathelicidins, the membrane permeability of *E. coli* caused by each peptide was examined with propidium iodide (PI), which penetrates bacteria with compromised membrane to stain nucleic acids. Flow cytometry analysis of PI uptake showed that all of the four peptides caused strong membrane damage within 30 min, resulting in percentages of bacteria cell populations staining PI-positive of 97.9%, 80.1%, 49.2%, and 97.5% for the BMAP-27, BMAP-34, mCRAMP, and LL-37 treatments, respectively (Figure 3). The vast majority of cells were membrane-damaged when treated with BMAP-27, BMAP-34, and LL-37 for 60 min, except the mCRAMP treatment, which caused only 64.5% PI-positive cells (Figure 3). Remarkably, BMAP-27 caused extremely rapid membrane damage with a significant PI uptake, affecting 95.4% of the population within 1 min, while LL-37, BMAP-34, and mCRAMP exhibited relatively slower membrane damage compared with BMAP-27, with 45.7%, 27.8%, and 14.5% PI-positive cells within 1 min, respectively (Figure 3).

**Figure 4:**
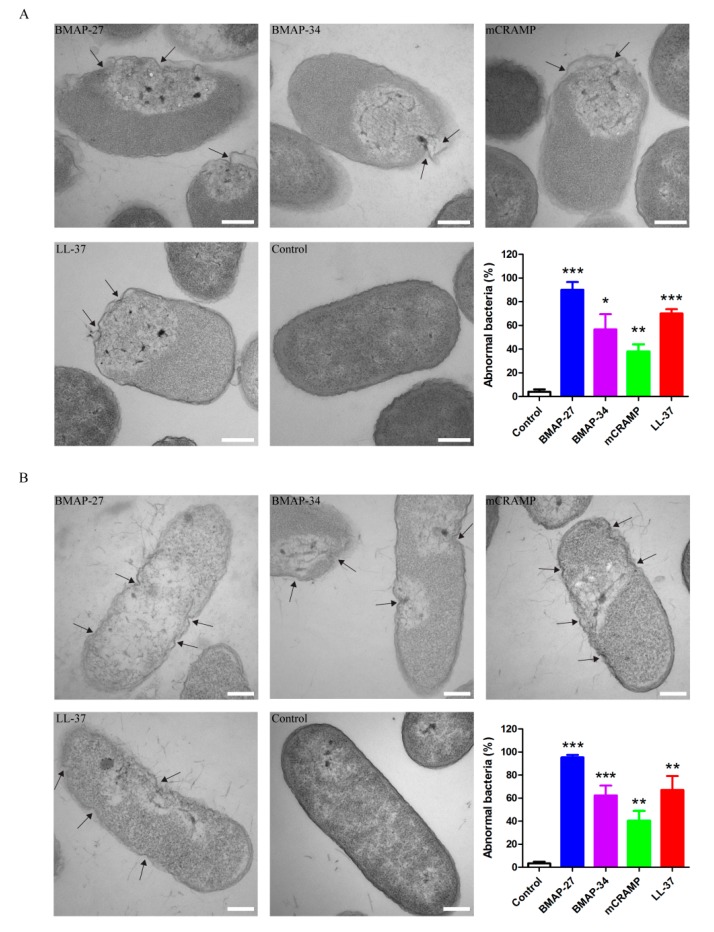
Mammalian cathelicidins disrupt the bacterial cell membrane. TEM of (**A**) *E. coli* and (**B**) *P. aeruginosa* in the mid-log phase at the treatment of BMAP-27, BMAP-34, mCRAMP, and LL-37 were carried out. All of the four peptides caused membrane damage, leading to varying degrees of lysed morphology and leakage of contents. The arrow indicates the sites of membrane damage. Scale bars = 200 nm. The percentages of abnormal cells after each peptide treatment are shown as mean ± SD from three independent counts and the number of cells for each count is 100 (*n* = 100), * *p* < 0.05, ** *p* < 0.01, *** *p* < 0.001.

Morphological changes of *E. coli* and *P. aeruginosa* after each peptide treatment were determined using transmission electron microscopy (TEM). As shown in Figure 4A, control cells of *E. coli* ATCC 29522 contained intact cell membranes and showed an even distribution of electron-dense. In contrast, the treatment of *E. coli* cells with each peptide led to severe morphological alterations and irregular surfaces. All of the four peptides caused membrane damage in a similar manner. The membranes exhibited dramatic fluctuations upon each peptide exposure, showing deep craters or open holes in their envelope, and the intracellular contents leaked out from the rupturing sites (Figure 4A). Approximately 90% of cells were abnormal after treatment with BMAP-27, whereas BMAP-34, mCRAMP, and LL-37 produced approximately 57%, 38%, and 70% of abnormal cells, respectively (Figure 4A). Likewise, control treatment of *P. aeruginosa* with no peptide exhibited intact electron-dense cells with a normal shape and smooth surface. After each peptide treatment, the membrane of *P. aeruginosa* showed varying levels of indentation and leakage of a large amount of contents (Figure 4B). Approximately 95% of cells were abnormal after treatment with BMAP-27, whereas BMAP-34, mCRAMP, and LL-37 produced approximately 62%, 40%, and 67% of abnormal cells, respectively (Figure 4B). Thus, although all of the four peptides induced similar membrane-damage patterns, the number of damaged cells of *E. coli* and *P. aeruginosa* is much higher after BMAP-27 treatment than after the exposure to the other three peptides.

### 2.3. BMAP-27, mCRAMP, and LL-37 Inhibit P. aeruginosa Biofilm Formation

**Figure 5:**
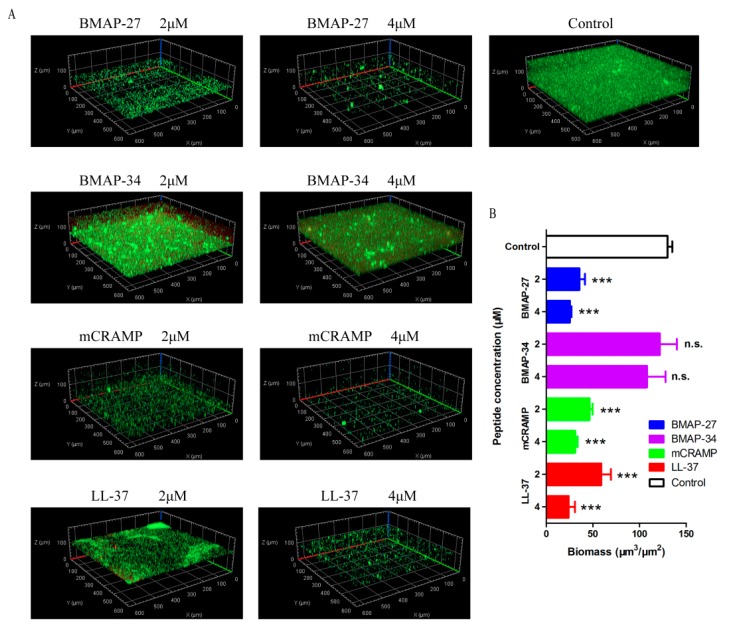
Mammalian cathelicidins BMAP-27, mCRAMP and LL-37 inhibit biofilm formation of *P. aeruginosa*. (**A**) Representative confocal microscope images of *P. aeruginosa* biofilm developed for 36 h at the treatment with the indicated concentrations of each peptide. (**B**) Quantification of biofilm biomass determined by analysis with COMSTAT. Values represent the mean ± SD from at least five image stacks, *** *p* < 0.001, n.s. = not significant.

Since it has been described that LL-37 prevents biofilm formation [14], other cathelicidins were further tested here for their antibiofilm properties against *P. aeruginosa*. Tridimensional biofilm formation after peptide treatment was investigated by confocal laser scanning microscopy. As shown in Figure 5A, *P. aeruginosa* forms a thick and confluent layer after 36 h. BMAP-27, mCRAMP, and LL-37 showed strong inhibitory effects on biofilm formation in *P. aeruginosa* (Figure 5A), but there was no significant difference in biofilm formation after BMAP-34 treatment at the concentrations of 2 μM or 4 μM compared with control biofilm (Figure 5A). To confirm these qualitative observations, quantitative analyses for biomass measurements were performed using COMSTAT software. As shown in Figure 5B, the median biomass of the *P. aeruginosa* biofilm was 129.9 μm^3^/μm^2^. BMAP-27, mCRAMP, and LL-37 were able to significantly (*p* < 0.001) decrease the biofilm biomass at both 2 μM or 4 μM, with biomass reaching a median of 35.6, 46.4, and 58.9 μm^3^/μm^2^ at the 2 μM, and 25.4, 31.0 and 24.1 μm^3^/μm^2^ at the 4 μM, respectively. However, BMAP-34 did not significantly reduce the biofilm biomass of *P. aeruginosa* (Figure 5B).

### 2.4. BMAP-34, mCRAMP, and LL-37 Potentiate the Immune Responses of Neutrophils

**Figure 6:**
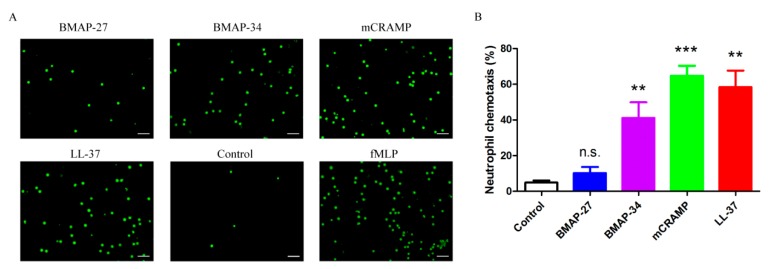
Mammalian cathelicidins BMAP-34, mCRAMP and LL-37 stimulate the chemotaxis of neutrophils. (**A**) Fluorescence microscopic analyses for the chemotactic properties of BMAP-27, BMAP-34, mCRAMP and LL-37. Human neutrophils (green) were seeded in the upper chamber of a 5 μm Transwell, migration was stimulated with each peptide in the lower chamber. Control, untreated neutrophils. N-Formyl-methionyl-leucyl-phenylalanine (fMLP) at 5 μM was used as positive control. Scale bars = 25 μm. (**B**) Neutrophil chemotaxis was calculated using the number of migrating neutrophils attracted by positive control fMLP as 100%. Values represent the mean ± SD from three independent assays, ** *p* < 0.01, *** *p* < 0.001, n.s. = not significant.

**Figure 7:**
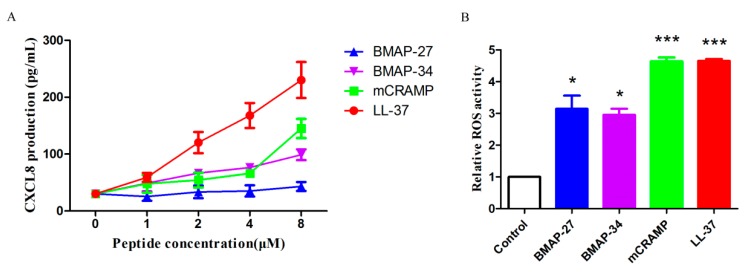
Mammalian cathelicidins affect the CXCL8 and ROS production. (**A**) CXCL8 production by human neutrophils in response to BMAP-27, BMAP-34, mCRAMP, and LL-37. (**B**) ROS production stimulated by the indicated concentrations of BMAP-27, BMAP-34, mCRAMP, and LL-37. Values represent the mean ± SD from three independent assays, * *p* < 0.05, *** *p* < 0.001.

It has been shown previously that LL-37 kills invading pathogens not only by directly perforating bacterial membranes but also by elevating the immune functions of neutrophils [7,8]. However, little is known about the immunomodulatory properties of other cathelicidins, including BMAP-27 and BMAP-34. Considering the potential applications of cathelicidins for the therapy of human bacterial diseases, their immunomodulatory properties for human neutrophils were investigated. The chemotactic abilities of the four cathelicidins for neutrophil migration were examined using a transwell assay. The results indicate that BMAP-34, mCRAMP, and LL-37 potently stimulated the chemotaxis of neutrophils, while BMAP-27 attracted rare neutrophil migration (Figure 6A). Notably, mCRAMP achieved even better chemotactic activity than LL-37 (Figure 6B). Furthermore, in neutrophils, BMAP-34, mCRAMP, and LL-37 significantly stimulated the production of CXCL8 (Figure 7A), which is an important chemotactic factor that induces neutrophil migration. Treatment of neutrophils with LL-37 resulted in the highest production of CXCL8 at concentrations of 1–8 μM, although BMAP-34 and mCRAMP also induced an increase in CXCL8 production in a dose-dependent manner (Figure 7A). BMAP-27 was found to have no apparent ability to stimulate neutrophil CXCL8 production (Figure 7A). The production of reactive oxygen species (ROS) in response to each peptide was then assessed using a ROS-sensitive fluorescent probe. The results indicated that the BMAP-27 and BMAP-34 treatment induced approximately three-fold greater ROS production in neutrophils compared with control cells (Figure 7B). Meanwhile, mCRAMP and LL-37 stimulated much more intracellular ROS formation, around 4.6-fold higher, compared to control cells (Figure 7B).

**Figure 8:**
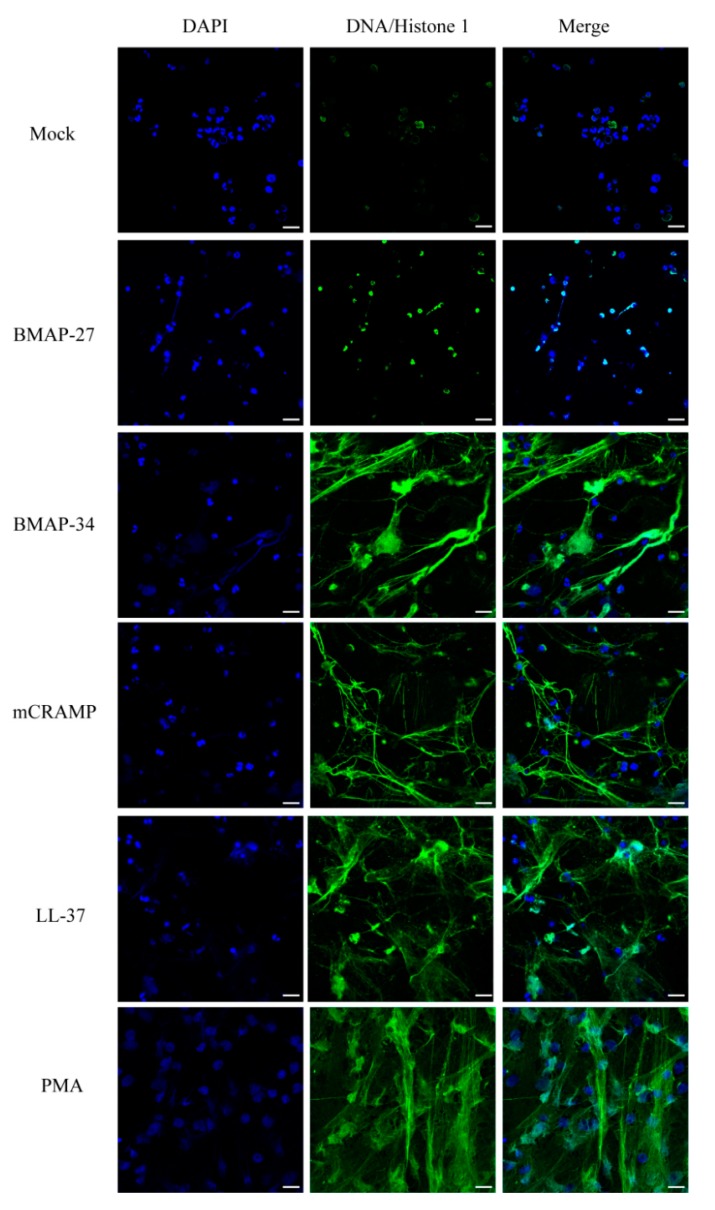
Mammalian cathelicidins promote the NETs formation. Fluorescence microscopic visualization of NETs formation stimulated by BMAP-27, BMAP-34, mCRAMP, and LL-37 for 4 h at 37 °C. NETs stimulated from human neutrophils were stained for DNA/histone 1 in green, nuclei in blue. Mock, unstimulated neutrophils. PMA, positive control. Scale bars = 20 μm.

It has been suggested that LL-37 and defensin hBD-1 could amplify the antibacterial properties of neutrophils by inducing the formation of neutrophil extracellular traps (NETs) [15,16], a defensive function used by neutrophils to entrap and kill pathogens. To investigate the abilities of other cathelicidins for NETs formation, human blood-derived neutrophils were treated with each peptide for 4 h to induce NETs formation and were then observed by confocal laser scanning microscopy. As shown in Figure 8, BMAP-34 could induce extensive NETs structures. Consistent with a previous finding [15], mCRAMP and LL-37 both induced a robust NETs formation. Unlike BMAP-34, mCRAMP, and LL-37, BMAP-27 did not induce appreciable NETs production over the spectrum of tested concentrations. These results indicated that compared with BMAP-27, the other three cathelicidins (BMAP-34, mCRAMP, and LL-37) could effectively promote the immune responses of neutrophils.

### 2.5. Combined Therapy with BMAP-27 and LL-37 Augments Clearance of Pulmonary P. aeruginosa Infection

The minimal inhibitory concentration (MIC) assay using the *P. aeruginosa* was performed to screen the efficacies of peptides, and a fractional inhibitory concentration (FIC) value of ≤ 0.5 was defined as indicating a synergistic effect for the combination of BMAP-27 and three other peptides. LL-37 was identified as having a synergistic effect with BMAP-27 (Table S1). The hemolytic activities of the four cathelicidins against human red blood cells were determined as an indication of their toxicity toward mammalian cells (Figure S1). None of the peptides exhibited notable hemolytic activity, with all being well under 20% hemolysis.

**Figure 9:**
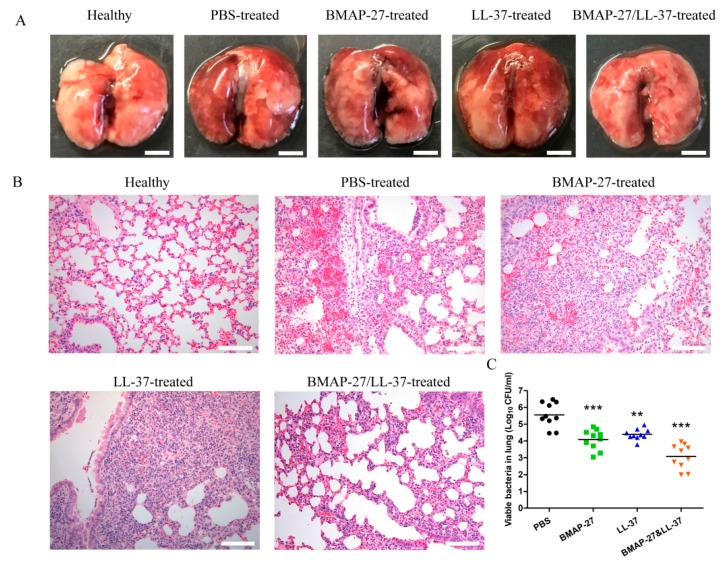
Combined therapy of BMAP-27 and LL-37 reduces the bacterial burden and organ injury in the mice after *P. aeruginosa* infection. Mice were intratracheally challenged with 3 × 10^6^ CFU of *P. aeruginosa* and then administered a single-dose treatment of BMAP-27, LL-37, the combination of BMAP-27 and LL-37, or PBS. At 24 h posttreatment, samples were harvested from the (**A**) lungs (scale bars = 4 mm) for (**B**) histopathological hematoxylin-eosin staining (scale bars = 100 μm) and (**C**) bacterial quantification. Values represent mean ± SD from 10 mice, ** *p* < 0.01, *** *p* < 0.001.

Here, the effect of bacterial clearance achieved by a combination therapy of BMAP-27 with LL-37 was determined *in vivo*. The influence of the combination of BMAP-27 and LL-37 on the organ injury and bacterial burden was examined in a murine model of respiratory infection. Mice (*n* = 10 per group) were infected with 3 × 10^6^ colony-forming units (CFUs) of *P. aeruginosa*, and received either BMAP-27 alone, LL-37 alone, BMAP-27 and LL-37 in combination, or phosphate-buffered saline (PBS) as a control. The therapeutic dosage *in vivo* was selected to minimize the toxic effects based on the lower hemolytic activity (<10%) of BMAP-27 and LL-37 at the concentration of 8 μM (Figure S1). At 24 h posttreatment, the lungs were harvested from different groups and examined. As shown in Figure 9A, overt inflammation injury in lung was evident in PBS-treated mice. This injury was reduced slightly in BMAP-27- or LL-37-treated mice. However, the lungs of combined BMAP-27/LL-37-treated mice appeared similar to those of normal mice (Figure 9A). The lungs of PBS-treated mice displayed hyperplasia of pulmonary alveolar epithelial cells, significantly widened alveolar ridges, severe blood stasis and hemorrhage, degeneration and necrosis of bronchiole epithelial cells and perivascular edema (Figure 9B). However, BMAP-27 or LL-37 treatment did not effectively alleviate these abnormalities associated with *P. aeruginosa* -induced tissue damage (Figure 9B). In contrast, only mild inflammatory cell infiltration was observed in the lung tissue of infected mice treated with the combination of BMAP-27 and LL-37.

The extent of organ injury is usually associated with the bacterial burden in mice. As shown in Figure 9C, both BMAP-27 and LL-37 rendered the bacterial burden in the lungs of the infected mice smaller than that in the PBS-treated controls (4.080 and 4.361 versus 5.556 log_10_ CFU/mL, respectively). However, BMAP-27 performed slightly more effectively than LL-37. Mice treated with the combination of BMAP-27 and LL-37 showed the lowest median bacterial burden (3.069 log_10_ CFU/mL) of any of the infected mice. These data indicated that the combination of BMAP-27 and LL-37 showed synergistic effects *in vivo*, and protected mice from *P. aeruginosa* infection by reducing bacterial burden and alleviating organ injury.

## 3. Discussion

As the first line of innate immune defenses, mammalian cathelicidins have rapidly captured attention as a novel therapeutic strategy, and efforts to bring them into clinical use are accelerating [3,17]. In this study, the antibacterial and immunomodulatory activities of four mammalian cathelicidins (BMAP-27, BMAP-34, mCRAMP, and LL-37) were systematically examined and compared. Additionally, the efficacy of the combination of BMAP-27 and LL-37 in an *P. aeruginosa* lung infection *in vivo* was examined.

Our data revealed that BMAP-27 has the highest antibacterial activity among these four cathelicidins. It rapidly destroyed the bacterial membrane integrity within only one minute and was highly effective at killing bacteria, including ESKAPE pathogens. This optimal bactericidal activity is likely due to its typical and facial amphiphilic structure. Amphiphilicity is the most striking feature of AMPs that have one cationic side and one hydrophobic side [18]. The distribution of basic and hydrophobic amino acids in BMAP-27 conforms to this typical characteristic, while those of BMAP-34, mCRAMP, and LL-37 are relatively more dispersed, causing the bactericidal activities of these peptides to be weaker than that of BMAP-27. In addition, charge and hydrophobicity are also important factors for the bactericidal activities of AMPs [19,20]. Given the net negative charge of bacterial surfaces containing lipopolysaccharides or lipoteichoic acids [21], the higher number of positive charges of BMAP-27 compared with the other three cathelicidins could increase its affinity for the bacterial membrane through electrostatic interaction. When adsorbed onto the bacterial membrane, the hydrophobic residues can further penetrate and disorganize the lipid tail region of the membrane [18,19]. Thus, the strong hydrophobicity of BMAP-27 accelerates membrane rupture and bacteria death.

*P. aeruginosa* growing in a biofilm state are very difficult to treat because of the considerable degree of protection from the host immune system attack or antibiotic therapy [22,23]. Of note, our data show that BMAP-27 is able to inhibit biofilm formation of *P. aeruginosa*, and it is the most efficacious antibiofilm agent among the four cathelicidins. LL-37 also displayed potent inhibition of biofilm formation by *P. aeruginosa*, which is consistent with the observations of a previous study [14]. It is known that AMPs has multiple mechanisms of action against biofilm formation [24]. For instance, LL-37 can prevent biofilm formation of *P. aeruginosa* possibly by down-regulating the transcription of the genes associated with the assembly of flagella and two major quorum-sensing systems, Las and Rhl [14]. Furthermore, the disruption or degradation of the membrane of biofilm-embedded cells by AMPs is also partly related to its antibacterial activity [24], such as BMAP-27 and LL-37. Interestingly, mCRAMP displayed a relatively weaker antibacterial activity compared with the other three cathelicidins, as the complete killing *P. aeruginosa* required a concentration of 8 μM. However, it exhibited a robust inhibition of biofilm formation only at the concentration of 2 μM, much like BMAP-27. This indicated that antibiofilm effect of mCRAMP is not mainly related to its permeabilization of the membrane of *P. aeruginosa*, but it may involve other potential mechanisms of action against biofilms formation. Whether mCRAMP interferes with the attachment of planktonic bacteria to surfaces, the aggregation of microcolonies, or the quorum-sensing systems needs further exploration in subsequent research. Currently, the fundamental structure and sequence requirements that govern the antibiofilm activity of AMPs are poorly understood. Although all of the four cathelicidins possess α-helical structure, and BMAP-34 does not possess antibiofilm activity against *P. aeruginosa*, perhaps the amino acids composition and flexibility of the peptide affects its interaction with molecules involved in various signaling pathways related to biofilm formation. Overall, it is worth noting that BMAP-27 has the most potent antibiofilm activity as well as antibacterial activity against to *P. aeruginosa*, thus highlighting its potential as a novel approach of controlling *P. aeruginosa* infection.

Recently, most of the claimed AMPs with available patent information referring to the therapeutic use were characterized not only as a potent antibacterial agent, but also as effective immunomodulators contributing to the bacterial clearance of the host [17]. For instance, LL-37 acts by regulating the production of inflammatory messengers, enhancing phagocytosis and intracellular killing of bacteria [3]. Interestingly, in our study, BMAP-34 and mCRAMP were shown to induce chemotaxis of neutrophils as well as LL-37, but they were also shown to stimulate neutrophils to increase CXCL8 production to varying degrees. We also found that all of the four cathelicidins stimulated the neutrophils to enhance the levels of ROS production, which could facilitate bacterial killing. A previous study has also demonstrated that, after stimulation with *S. aureus*, peritoneal neutrophils from mCRAMP-deficient mice produce significantly lower levels of ROS than the cells from WT mice, which together with our study underscore the importance of the cathelicidins in the process of ROS production [25]. LL-37 has been shown to facilitate NETs formation, which is a novel antibacterial defense strategy against pathogenic bacteria [15,26]. We also found that BMAP-34 and mCRAMP exhibit the NETs-inducing effect on neutrophils similar to that of LL-37. Notably, a recent study impressively demonstrated that formation of NETs could act as an effective barrier, confining *P. aeruginosa* to the external environment and blocking dissemination into other important organs such as the brain [27]. Our finding of the NETs-inducing effects of LL-37, BMAP-34, and mCRAMP suggests that they may have significant implications for effectively controlling further dissemination and promoting the clearance of *P. aeruginosa* infection. The modulation of AMPs on the immune system involves interacting with multiple immune receptors and subsequent signaling pathways of immune responses [7]. Our data showed that the facial amphiphilic structure of BMAP-27 was beneficial for its bactericidal activity, but seems to have less effect on its immunomodulatory property. Meanwhile, the distribution of hydrophobic and positively charged residues in BMAP-34, mCRAMP, and LL-37 are more dispersed, which may promote the interaction with certain immune receptors and modulate the immune response of neutrophils.

Given the outstanding bactericidal and antibiofilm properties of BMAP-27, and the immune promotion effect of LL-37, the principal interest in our study was whether a combinatorial strategy can effectively enhance the clearance of *P. aeruginosa* pulmonary infection. Although the marked synergistic effect of other mammalian cathelicidins against bacterial pathogens has been described *in vitro* [28], the *in vivo* effect of combined cathelicidins has not been determined. Mark et al. has demonstrated that AMPs synergistically contribute to defense against pathogens *in vivo* using CRISPR gene editing to delete ten known immune-inducible AMPs from fruit flies [11], thus implying the feasibility of combination therapy. Beaumont et al. observed that the administration of synthetic LL-37 promoted the upregulation of the early neutrophil response and enhanced *P. aeruginosa* clearance *in vivo* [29]. Our data indicated that, compared with LL-37, BMAP-27 was slightly more potent *in vivo* bactericidal activity by reducing the burden of *P. aeruginosa* in the lung. Moreover, the combination of BMAP-27 and LL-37 displayed an impressive therapeutic efficacy that was superior to that of BMAP-27 or LL-37 alone for clearance of *P. aeruginosa* infection. Taken together, these results suggest a promising medical application by exploiting a combinatorial strategy involving BMAP-27 and LL-37 to treat bacterial infections. This approach will allow the use of a lower therapeutic dose of each peptide, thus minimizing the risk of adverse effects. Beyond the direct membrane-lytic mechanism, many AMPs are also able to exert intracellular inhibitory activities, including the targeting of nucleic acid biosynthesis, protein-folding, and cell division [30,31,32]. The combination of multiple targeting and rapid bactericidal activities would essentially minimize the chance of AMPs resistance development in the bacteria [33]. Our future research will focus on identifying the appropriate synergistic combinations of AMPs with different beneficial properties as valid cocktail agents against bacterial infections.

## 4. Materials and Methods

### 4.1. Bacterial Strains and Growth Conditions

*Escherichia coli* ATCC 25922, *Pseudomonas aeruginosa* ATCC 27853, *Acinetobacter baumannii* ATCC 19606, and *Klebsiella pneumoniae* ATCC 700603 were cultured in Luria-Bertani (LB, Difco Laboratories, Detroit, MI, USA) medium. *Staphylococcus aureus* ATCC 29213 and *Enterococcus faecium* ATCC 700221 were cultured in a brain heart infusion (BHI, BD Biosciences, Franklin Lakes, NJ, USA) medium. All of the strains were routinely grown at 37 °C.

### 4.2. Bioinformatics Analysis

The net charge and the hydrophobic percentage of each peptide were obtained from the APD3 database [34]. Helical wheel representation of the helix of each peptide was generated using HeliQuest [35]. 3D structures of BMAP-27 and LL-37 peptides were obtained from the RCSB Protein Data Bank [36]. The 3D structures of BMAP-34 and mCRAMP were predicted using I-TASSER [37]. The structure of each peptide was presented using PyMOL [38].

### 4.3. Bactericidal Assays

The bactericidal activities of BMAP-27, BMAP-34, mCRAMP, and LL-37 (GL peptide Inc., Shanghai, China, purity > 98%) were analyzed as described previously [39]. Briefly, each strain was grown at 37 °C until the mid-log phase and washed in PBS three times. Bacterial suspensions (1 × 10^6^ CFU/mL) in PBS were incubated with peptides at different concentrations for 2 h. Serial dilutions of the bacteria were plated on LB or BHI agar, and incubated at 37 °C. After 24 h, the number of bacterial colonies was counted. 

### 4.4. PI Uptake Assays

The membrane permeability of *E. coli* induced by different peptides was measured by detection of PI uptake as described previously [40]. A mid-logarithmic growth-phase culture of *E. coli* ATCC 25922 was washed three times and diluted in PBS (pH 7.4) to 1 × 10^6^ CFU/mL. The bacteria were incubated with a final concentration of 4 μg/mL of PI (Thermo Fisher, Waltham, MA, USA) for 15 min, after which they were mixed with 4 μM of different peptides. PI fluorescence in the treated cells was quantified for 1 h at the indicated time points with flow cytometry using a BD Accuri C6 Flow Cytometer (BD Biosciences, Franklin Lakes, NJ, USA), and further analyzed with FlowJo (Treestar, Ashland, OR, USA).

### 4.5. TEM Experiment

*E. coli* ATCC 25922 and *P. aeruginosa* ATCC 27853 were grown at 37 °C until mid-log phase. The cultures were washed three times and diluted in PBS to 2 × 10^8^ CFU/mL and exposed to 4 μM of each peptide in PBS for 30 min at 37 °C. The bacteria without any peptide treatment were used as control. After 1 h, the samples were fixed with 2.5% glutaraldehyde overnight and then post-fixed with 1% OsO_4_ for 2 h at 4 °C. Samples were dehydrated in a stepwise acetone and imbedded in resin. After resin polymerization at 70 °C for two days, 65–70 nm sections were cut by a UC6 ultra-microtome (Leica Microsystems, Vienna, Austria) and stained with uranyl acetate and lead citrate before being visualized by a transmission electron microscope H-7650 (Hitachi, Tokyo, Japan). The ratios of morphologically abnormal cells in each sample were calculated in three independent counts, and the total number of cells for each count is 100.

### 4.6. Biofilm Inhibition Assay

The biofilm inhibition assay was performed as described previously [41]. *P. aeruginosa* ATCC 27853 was grown at 37 °C until the mid-log phase. In order to assess biofilm inhibition ability by the peptides, 180 μL of bacterial suspension (5 × 10^5^ CFU/mL) in LB medium and 20 μL of different peptides at final concentrations of 2 μM and 4 μM were added into the glass-bottom culture dishes, and the mixtures were incubated for 36 h at 37 °C. The floating bacteria were removed by gentle washing with 0.85% physiological saline. For confocal microscopic observation, the biofilms were stained with LIVE/DEAD BacLight Bacterial Viability kit (Thermo Fisher) according to manufacturer’s instructions. Fluorescent images were acquired using the LSM 800 confocal laser scanning microscope (Carl Zeiss, Oberkochen, Germany). Quantification of biofilm was analyzed for the biomass using the COMSTAT version 2.1 [42].

### 4.7. Isolation of Human Neutrophils

Human neutrophils were isolated from freshly collected blood from healthy donors that had provided written informed consent in accordance with the declaration of Helsinki (2013) of the World Medical Association and approved by the ethics committee of the of Harbin Veterinary Research Institute of the Chinese Academy of Agricultural Sciences. Cells were isolated using the PolymorphPrep system (Axis Shield, Dundee, UK) according to the manufacturer’s protocol and resuspended in RPMI 1640 medium.

### 4.8. Neutrophil Chemotaxis Assay

Neutrophil migration was evaluated as described previously [43]. Peptides were diluted in RPMI 1640 medium to a final concentration of 1 μM and placed in the lower compartment of 5-μm-pore transwell plates (Corning Life Sciences, Acton, MA, USA). As a positive control, N-Formyl-methionyl-leucyl-phenylalanine (fMLP, Sigma-Aldrich, St. Louis, MO, USA) at 5 μM was added in the lower chamber. Neutrophils (1 × 10^6^ cells) were labeled with CellTracter Green (Invitrogen, Carlsbad, CA, USA) and placed in the upper compartment of the chamber. Following incubation for 45 min, the neutrophil migration was monitored by fluorescence microscopy (Zeiss, Oberkochen, Germany) and the percentage of chemotactic cells was calculated using the number of migrating cells in the lower compartment of positive control as 100%.

### 4.9. CXCL8 Production Assay

Neutrophils (3 × 10^6^ cells/well) in 300 μL of RPMI were incubated with various concentrations (0–8 μM) of peptides for 6 h at 37 °C under 5% CO_2_. The supernatants were collected and the amount of CXCL8 chemokine was quantified by an enzyme-linked immunosorbent assay kit (Sigma-Aldrich) in accordance with the manufacturer’s instructions.

### 4.10. ROS Measurement

ROS production was determined using the 2′,7′-dichlorofluorescein diacetate (DCFDA), a fluorescence indicator of intracellular ROS as described previously [44]. Neutrophils (2 × 10^5^ cells) were either untreated (as control) or treated 2 μM of each peptide for 1 h. Then, the cells were stained with 5 μM DCFDA (Sigma-Aldrich) in the dark. After staining for 20 min, cells were washed with PBS three times. The intensity of green fluorescence was read using an EnVision Multilabel Reader (PerkinElmer, Plymouth, UK) with excitation at 485 nm and emission at 520 nm to measure the ROS production. Relative ROS activity was calculated as the value for the treatment group/control group.

### 4.11. NETs Formation Assay

The NETs formation assay was performed as described previously [45]. Neutrophils (2 × 10^5^ cells) were seeded into the glass-bottom culture dish pre-coated with poly-l-lysine, then exposed to 6 μM peptides for 4 h. PMA at 25 nM as a positive control was used to stimulate the NETs formation. The cells were fixed with 4% paraformaldehyde for 10 min and blocked with 3% bovine serum albumin (Sigma-Aldrich) in 0.2% Triton X-100 for 45 min. Then, cells were incubated by mouse antiDNA/Histone 1 Antibody (Merck Millipore, Darmstadt, Germany) at 4 °C overnight, and stained with Alexa Fluor 488-conjugated goat antimouse IgG and 4’6-diamidino-2-phenylindole (DAPI) (Sigma-Aldrich). Fluorescence images were acquired using the LSM 800 confocal microscope (Carl Zeiss, Oberkochen, Germany).

### 4.12. Hemolysis Assay

Hemolysis was performed by incubating a 4% (w/v) human red blood cell suspension in PBS (pH 7.4) with various concentrations (0–64 μM) of peptides for 1 h at 37 °C. Total hemolysis (A_triton_) was obtained by adding 0.2% Triton X-100 to the cell suspension. The samples were then centrifuged and the supernatant absorbance was read at 415 nm. The percentage of hemolysis was calculated using the following formula: hemolysis % = [(*A*_sample_ − *A*_PBS_)/(*A*_triton_ − *A*_PBS_)] × 100.

### 4.13. Determining MIC and FIC

MIC values of peptides were determined by the twofold serial dilution method in 96-well flat-bottomed microtitration plates at a final concentration of 1 × 10^6^ CFU/mL. MIC value was defined as the lowest concentration of peptides that inhibited visible growth. The interaction between BMAP-27 and other three cathelicidins against *P. aeruginosa* was evaluated following the checkerboard method [46]. FIC was used to evaluate the effectiveness of the combinations. The formula for calculation of the FIC index was FIC index = MIC_A in combination_/MIC_A alone_ + MIC_B in combination_/MIC_B alone_, where A is BMAP-27 and B is one of the other cathelicidins. The interaction was defined as synergistic if the FIC index was <1.0, additive if the FIC index was 1.0, sub-additive if the FIC index was between 1.0 to <2.0, indifferent if the FIC index was 2, and antagonistic if the FIC index >2. Synergy was further sub-classified as marked (FIC index, <0.50) and weak (FIC index, between 0.50 to <1.0) [46,47].

### 4.14. Murine Infection Model Treatment

The animal experiments were carried out based on the protocol (HVRI-IACUC-2019-179) approved by the ethics committee of the Harbin Veterinary Research Institute of the Chinese Academy of Agricultural Sciences. *P. aeruginosa* ATCC 27853 cells were grown to the mid-logarithmic phase, washed with PBS three times, and diluted in the same buffer to 6 × 10^7^ CFU/mL. Mouse infection was performed on the murine model described previously [48]. Briefly, six-week-old specific-pathogen-free female BALB/c mice were anesthetized by isoflurane inhalation and instilled intratracheally with 50 μL bacterial suspension (3 × 10^6^ CFU). One hour after bacterial exposure, 50 μL of 8 μM BMAP-27, 8 μM LL-37, the combination of 4 μM BMAP-27 and 4 μM LL-37, or PBS was instilled intratracheally into mice. To study the effectiveness of bacterial dissemination, mice were euthanized for 24 h after bacterial exposure, and their lungs were removed and homogenized. Homogenization or serial dilution of samples were placed on LB ager plates. The number of CFU was counted after incubation overnight at 37 °C and was expressed as CFU/g for lung tissues. In order to analyze the histopathologic changes of lung tissues, mice were euthanized 24 h after bacterial infection, and their lungs were quickly removed and fixed overnight in 4% paraformaldehyde solution. Then, the lungs were dehydrated, embedded in paraffin, sectioned at 4 μm, stained with hematoxylin and eosin, and microscopically examined for histopathologic changes.

### 4.15. Statistical Analysis

Statistical analyses were performed using GraphPad Prism version 7.0 (GraphPad Software, San Diego, CA, USA). Data were analyzed by performing Student’s *t*-test or one-way analyses of variance. *P*-values less than 0.05 were considered to be statistically significant (* *p* < 0.05; ** *p* < 0.01; *** *p* < 0.001).

## Supplementary Materials

The following are available online at https://www.mdpi.com/1422-0067/21/5/1871/s1, Figure S1: Hemolytic activity of mammalian cathelicidins. 100% hemolysis was established using 0.2% Triton X-100 (ATriton). Percentage hemolysis was calculated as follows: hemolysis% = [(ASample − APBS) / (ATriton − APBS)] × 100. Values represent the mean ± SD from three independent assays, Table S1: MIC and FIC indices of BMAP-27 in combination with other peptides against P. aeruginosa.

## Abbreviations

AMPs	antimicrobial peptides
BMAP-27	bovine myeloid antimicrobial peptides-27
BMAP-34	bovine myeloid antimicrobial peptides-34
mCRAMP	murine cathelicidin-related antimicrobial peptide
LL-37	lys lys-37
MDR	multidrug-resistant
3D	three-dimensional
PDB	protein data bank
I-TASSER	iterative threading assembly refinement
APD3	antimicrobial peptide database 3
MBC	minimal bactericidal concentration
MIC	minimal inhibitory concentration
FIC	fractional inhibitory concentration
ATCC	American type culture collection
PI	propidium iodide
TEM	transmission electron microscopy
fMLP	N-Formyl-methionyl-leucyl-phenylalanine
CXCL8	chemokine (C-X-C motif) ligand- 8
ROS	reactive oxygen species
hBD-1	human beta defensins-1
NETs	neutrophil extracellular traps
CFU	colony-forming unit
PBS	phosphate-buffered saline
LB	Luria-Bertani
BHI	brain heart infusion
DCFDA	2′,7′-dichlorofluorescein diacetate
DAPI	4’6-diamidino-2-phenylindole
BALB	Bagg’s albino
ESKAPE	*Enterococcus faecium, Staphylococcus aureus, Klebsiella pneumoniae, Acinetobacter baumannii, Pseudomonas aeruginosa*, and Enterobacter species
