# Supplementary material for: Differential Abilities of Mammalian Cathelicidins to Inhibit Bacterial Biofilm Formation and Promote Multifaceted Immune Functions of Neutrophils
Source: Int J Mol Sci. 2020 Mar 9;21(5):1871. doi: 10.3390/ijms21051871 (PMC7084556; doi:10.3390/ijms21051871)
Supplement: Supplementary file 1 [file ijms-21-01871-s001.pdf]

Table S1. MIC and FIC indices of BMAP-27 in combination with other peptides against *P. aeruginosa*.

Peptide	MIC (μM [Fold Change])		FIC index
	Alone	Combination	
BMAP-27	1	0.25 (4)	0.5
LL-37	4	1 (4)	
BMAP-27	1	1 (1)	1.25
mCRAMP	8	2 (4)	
BMAP-27	1	0.5 (2)	0.75
BMAP-34	4	1(4)	

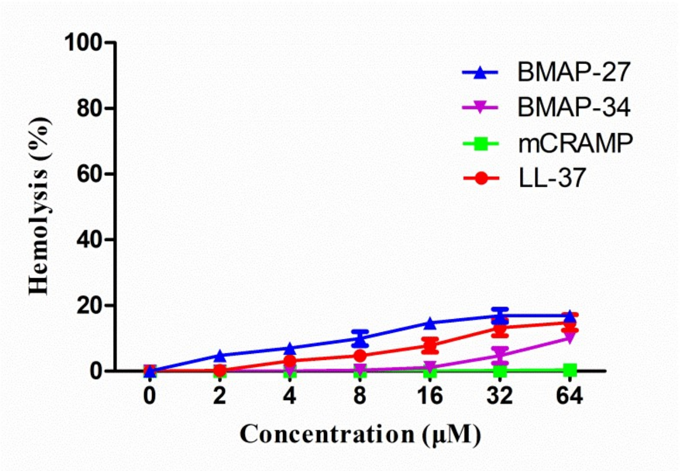

Figure S1. Hemolytic activity of mammalian cathelicidins. 100% hemolysis was established using 0.2% Triton X-100 (A_{Triton}). Percentage hemolysis was calculated as follows: hemolysis% = [(A_{Sample} − A_{PBS}) / (A_{Triton} − A_{PBS})] × 100. Values represent the mean ± SD from three independent assays.
